# Differential Effect of Smoking on Gene Expression in Head and Neck Cancer Patients

**DOI:** 10.3390/ijerph15071558

**Published:** 2018-07-23

**Authors:** Alexandra Iulia Irimie, Cornelia Braicu, Roxana Cojocneanu, Lorand Magdo, Anca Onaciu, Cristina Ciocan, Nikolay Mehterov, Diana Dudea, Smaranda Buduru, Ioana Berindan-Neagoe

**Affiliations:** 1 Department of Prosthetic Dentistry and Dental Materials, Division Dental Propaedeutics, Aesthetic, Iuliu Hatieganu University of Medicine and Pharmacy, Cluj-Napoca, 23 Marinescu Street, Cluj-Napoca 40015, Romania; irimie.alexandra@umfcluj.ro (A.I.I.); ddudea@umfcluj.ro (D.D.); 2 Research Center for Functional Genomics and Translational Medicine, Iuliu Hatieganu University of Medicine and Pharmacy, 23 Marinescu Street, Cluj-Napoca 40015, Romania; cornelia.braicu@umfcluj.ro (C.B.); roxana.petric@umfcluj.ro (R.C.); lorand.magdo@gmail.com (L.M.); ioana.neagoe@umfcluj.ro (I.B.-N.); 3 MEDFUTURE-Research Center for Advanced Medicine, University of Medicine and Pharmacy Iuliu Hatieganu, 23 Marinescu Street, Cluj-Napoca 40015, Romania; anca.onaciu@umfcluj.ro (A.O.); cristina.ciocan@umfcluj.ro (C.C.); 4 Department of Medical Biology, Faculty of Medicine, Medical University-Plovdiv, 15-А Vassil Aprilov Blvd., Plovdiv 4000, Bulgaria; ni_ki82@abv.bg; 5 Technological Center for Emergency Medicine, 15-А Vassil Aprilov Blvd., Plovdiv 4000, Bulgaria; 6 Prosthetics and Dental Materials, Faculty of Dental Medicine, Iuliu Hatieganu University of Medicine and Pharmacy, Cluj-Napoca, 32 Clinicilor Street, Cluj-Napoca 400006, Romania; 7 Department of Functional Genomics and Experimental Pathology, The Oncology Institute Ion Chiricuta, Republicii 34th Street, Cluj-Napoca 400015, Romania

**Keywords:** head and neck squamous cell carcinomas, smoking, TGCA data, gene expression data, survival rate

## Abstract

Smoking is a well-known behavior that has an important negative impact on human health, and is considered to be a significant factor related to the development and progression of head and neck squamous cell carcinomas (HNSCCs). Use of high-dimensional datasets to discern novel HNSCC driver genes related to smoking represents an important challenge. The Cancer Genome Atlas (TCGA) analysis was performed in three co-existing groups of HNSCC in order to assess whether gene expression landscape is affected by tobacco smoking, having quit, or non-smoking status. We identified a set of differentially expressed genes that discriminate between smokers and non-smokers or based on human papilloma virus (HPV)16 status, or the co-occurrence of these two exposome components in HNSCC. Kyoto Encyclopedia of Genes and Genomes (KEGG) pathways classification shows that most of the genes are specific to cellular metabolism, emphasizing metabolic detoxification pathways, metabolism of chemical carcinogenesis, or drug metabolism. In the case of HPV16-positive patients it has been demonstrated that the altered genes are related to cellular adhesion and inflammation. The correlation between smoking and the survival rate was not statistically significant. This emphasizes the importance of the complex environmental exposure and genetic factors in order to establish prevention assays and personalized care system for HNSCC, with the potential for being extended to other cancer types.

## 1. Introduction

Head and neck squamous cell carcinomas (HNSCCs) represent a preventable pathology which continues to be an important factor of morbidity with high mortality rates at global level [1,2], with over 600,000 new cases detected each year [3,4], and a mortality rate of around 50% [5]. HNSCCs have as common localizations the oral cavity, oropharynx, hypopharynx, and larynx [1,5,6].

An important aspect in prevention and treatment is related to genetic and environmental components [2,7,8]. While the acquired genetic factors cannot be controlled in their early steps of mutation accumulation, environmental exposure can significantly affect the pathogenesis and the prognosis of these patients [8,9]. The major environmental components are tobacco smoking, betel quid chewing, alcohol consumption, and poor oral hygiene and infections, alongside other specific dietary habits or specific pollutant exposure [6,7,10,11]. The totality of risk factors are integrated into the exposome [12], this being an important step in the evaluation of internal and external exposure, generally related to the co-occurrence of multiple toxic environmental agents [13]. Usually, the effect of co-occurrence is much more dramatic than that of the single exposure [13].

Microarray data are provided by large consortium programs such as The Cancer Genome Atlas (TCGA), offering new possibilities and a better understanding of the role of genes in different cancers [2,14]. Analysis of altered gene expression signatures is used in a wide range of pathologies for achieving relevant information with prognostic value [15]. The altered transcriptomic signatures can be integrated in multiple biological pathways, thus leading to a better comprehension of the fundamental mechanisms that are related to pathological processes influenced by smoking, and can be used for the selection of optimal therapy [16,17].

We anticipate that smoking affects molecular mechanisms, including transcriptomic patterns; therefore, we performed a TGCA data analysis in order to identify specific transcriptomic alterations in smoking versus non-smoking patients with head and neck cancer. These data provide a unique opportunity to study the potential oncogenic role of tobacco smoking; furthermore, based on the human papilloma virus (HPV)16 status and its effects on gene expression patterns, we intend to find whether these patients have different gene expression landscapes for the selected exposome components, or for the co-occurrence of these two important components of the exposome.

## 2. Materials and Methods

### 2.1. The Cancer Genome Atlas Gene Expression Data for Head and Neck Squamous Cell Carcinomas

The data were downloaded from the University of California Santa Cruz (USCS) Genome Browser [18] as a gene expression data matrix containing log_2_ transformed, normalized gene expression data for 519 tumor and 43 normal tumor-adjacent tissue samples (from 13 females and 30 males) and generated by RNA sequencing. Table 1 contains the demographic and clinical characteristics of the 519 patients that the tumor tissues were collected from, including clinical stages, information related to TNM (T: tumor, N: node, Mx: metastases), smoking history, and tumor localization.

The initial differential expression analysis was performed on the entire group of samples, namely 519 HNSCC tumors and 43 tumor-adjacent normal tissues. Further analyses were conducted on groups of patients divided according to their smoking status at the moment of sample collection: current smokers, quitters (also known as reformed smokers or ex-smokers), and never smokers.

Differential expression analysis was performed using the Gene Spring GX v.13.0 software from Agilent Technologies (Santa Clara, CA, USA), using the “volcano plot” module, and applying a fold change cut-off of ±2, moderated t-test and false discovery rate (FDR) correction. The bioinformatics analyses for differential expression were performed in the case of tumor tissue (*n* = 519) versus normal tissue (*n* = 43), and different comparisons were made based on smoking status, such as current smoking (*n* = 174) versus never smoked (*n* = 118), having quit smoking (*n* = 209) versus never smoked (*n* = 118), and finally for currently smoking (*n* = 174) versus having quit smoking (*n* = 209).

### 2.2. Molecular Classification for Gene Expression

Signature was performed using different online tools, such as String version 10.5 [19], Kyoto Encyclopedia of Genes and Genomes (KEEG) pathways [20], PantherDB [21] and miRnet data base [22].

### 2.3. Survival Analysis

Kaplan–Meier survival analysis was performed to investigate the survival distribution between selected groups based on the smoking status using Graph Pad Prism software (Version 6, Graph Pad software Inc., San Diego, CA, USA). A plot of the Kaplan–Meier analysis with the selected groups based on smoking status was performed.

## 3. Results

### 3.1. Differential Gene Expression in Tumor Tissues Versus Normal Tissues for Head and Neck Squamous Cell Carcinomas

**Figure 1:**
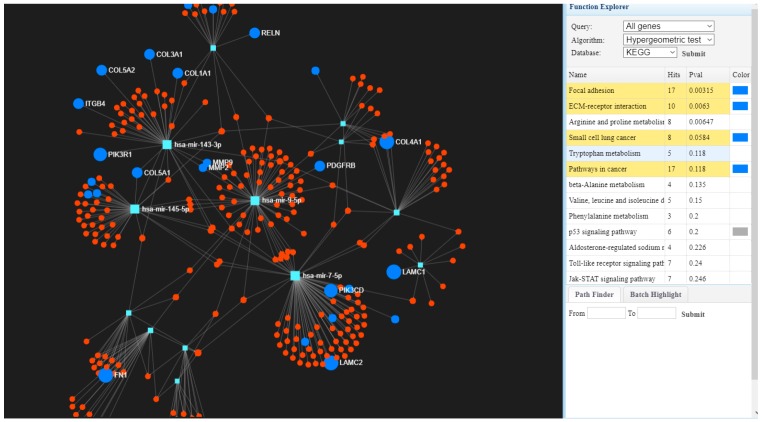
The interconnected genes with specific miRNAs using miRnet [22] involved in focal adhesion, extracellular matrix (ECM)–receptor interaction, or pathways in cancer interconnected with targeting microRNAs (miRNAs).

Global gene expression was evaluated in tumor tissues (*n* = 519) versus normal tissues (*n* = 43), where we identified 1216 upregulated genes and 1751 downregulated genes considering as cut-off the fold change (FC) value of ±2 and *p*-value ≤0.001 (Benjamini–Hochberg correction). Based on the KEGG classification, most of the upregulated genes belong to the extracellular matrix ECM–receptor interaction, focal adhesion, the PI3K (Phosphoinositide 3-kinase) –Akt (Protein kinase B) signaling pathway, or cell cycle regulation, while downregulated genes are involved in altered pathways belonging to drug metabolism cytochrome P450, chemical carcinogenesis and metabolism of xenobiotic by cytochrome P450. We also generated a map describing interconnections of the altered genes with specific targeting miRNAs (microRNAs) in Figure 1, using the miRnet database.

### 3.2. Differential Gene Expression Levels in Smokers Compared with Non-Smokers or Ex-Smokers in Head and Neck Squamous Cell Carcinomas

**Figure 2:**
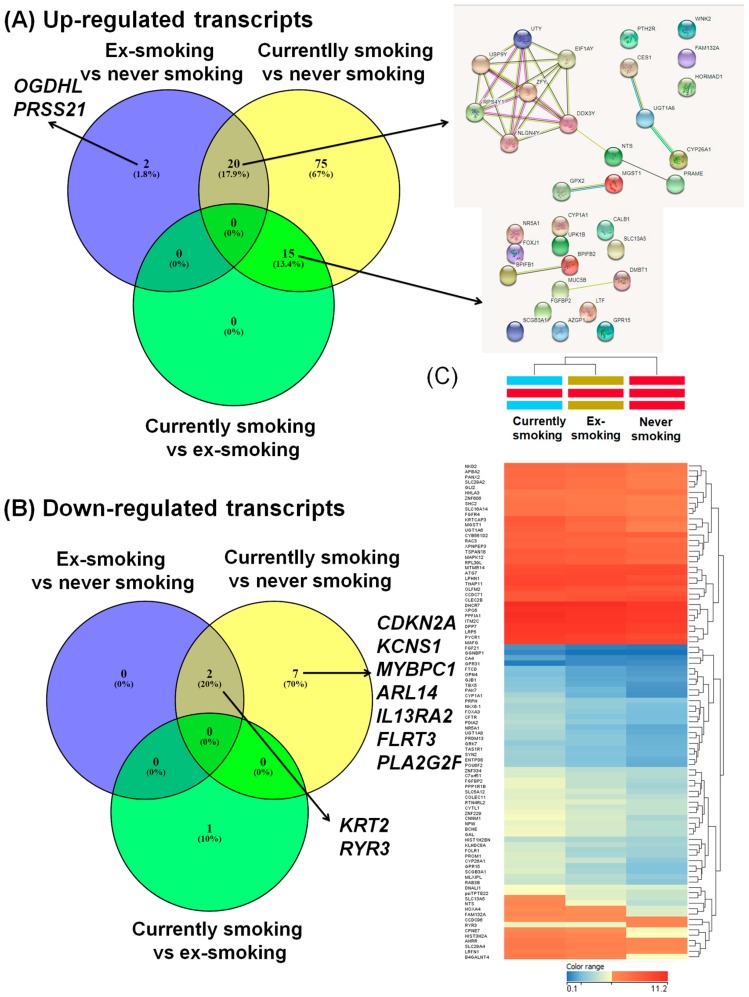
Venn diagram used for overlapping the altered gene expression pattern in the case of the three studied groups. Common and specific gene expression signatures for the three groups of HNSCC (head and neck squamous cell carcinomas) patients: non-smokers, ex-smokers, and smokers. (**A**) For the case of overexpressed genes, 20 common genes from non-smokers vs. smokers and ex-smokers vs. smokers, integrated as network using String, version 10.5) [19]; (**B**) The case of downregulated genes; (**C**) Heat maps for the expression level for the three HNSCC patient groups (current smokers, quitters, non-smokers), in dark blue being presented the downregulated genes and in red those overexpressed genes, generated using Gene Spring version 13.0.

In order to evaluate the transcriptomic alterations related to smoking in the tumor tissues, we performed multiple comparisons. In the first analysis we compared the gene expression pattern between current smokers and patients who had never smoked, identifying 119 altered transcripts (9 downregulated genes and 110 upregulated) (Table 2). The second analysis compared the gene expression pattern between ex-smokers and patients who had never smoked, revealing 24 altered transcripts (22 upregulated genes and 2 downregulated). The String network is presented in Figure 2A, for the 20 common upregulated genes for the group of ex-smokers vs. never smoked, and current smokers vs. never smoked, respectively. The third analysis compared the gene expression pattern between current smokers versus those who had quit, revealing 15 overexpressed genes, with the String network displayed in Figure 2A; the genes are not connected in specific networks. Figure 2B shows downregulated genes, presented as Venn diagrams for the analyzed groups (current smokers, ex-smokers, non-smokers), and emphasizes a signature in the ex-smokers group, revealing a panel of genes with an altered expression level event after quitting smoking in HNSCC patients.

In Figure S1, the String Network was generated for the altered signature in the case of smoking versus non-smoking, showing that most genes are involved in the metabolism of xenobiotics by cytochrome P450.

### 3.3. Molecular Classification for Altered Gene Expression Signature in Smoking versus Never Smoking Head and Neck Squamous Cell Carcinomas Patients

In order to perform the classification of the 119 altered genes in smoking versus never smoking HNSCC patients we used different online tools, such as String database [19] KEGG pathways [20] and PantherDB [21].

**Figure 3:**
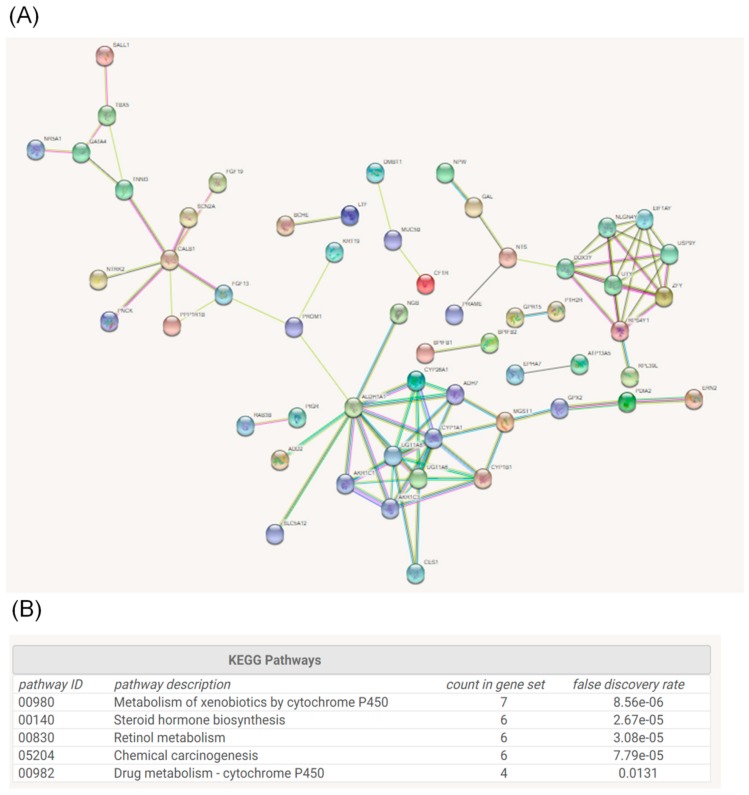
Gene network generated using Sting program; (**A**) Network of the interconnected genes; (**B**) KEGG (Kyoto Encyclopedia of Genes and Genomes) classification based on the altered signature in smoking versus never-smoking HNSCC patients.

The String network for the modified gene expression is presented in Figure 3A, and the KEGG classification in Figure 3B. In the KEGG pathways classification, most of the genes are related to cellular metabolism, emphasizing the activation of detoxification pathways, chemical carcinogenesis, or drug metabolism. Gene ontology classification based on molecular function and biological processes is presented in Table 3.

### 3.4. Effect of Smoking on Head and Neck Squamous Cell Carcinomas Stages

**Figure 4:**
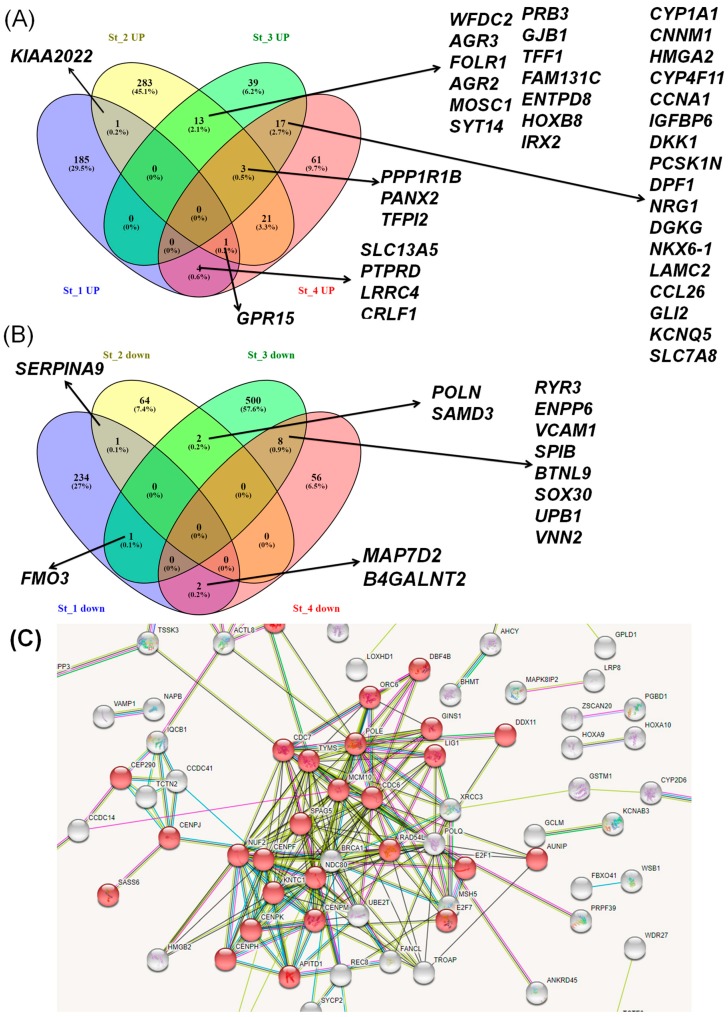
Venn diagram overlapping the (**A**) overexpressed and (**B**) downregulated genes based on a specific analysis of current smokers versus never smokers with HNSCC stage 1, 2, 3, and 4; (**C**) String Network for the case of downregulated genes in current smokers versus never smokers in HNSCC stage 1, where the genes involved in cell cycle regulation are highlighted in red.

To evaluate potential alteration of gene expression, specific for early stages to advanced tumor status, we compared the molecular profiles of tumor tissue in smoking versus non-smoking, according to tumor stages. Results from the computation of specific gene expressions for current smokers versus never smokers on HNSCC stages identified stage-specific gene expression signatures. The data presented as Venn diagrams illustrate the common and different overexpressed and downregulated genes (Figure 4A,B), while the network created using the String tool for the downregulated genes in the case of current smokers versus never smokers on HNSCC stage 1, revealed 32 genes involved in cell cycle regulation (Figure 4C).

To address the probable alteration of gene expression as an effect of advanced tumor status, we restricted the gene ontology classification of smoking versus non-smoking only for stage 1 tumors. Gene ontology based on the altered signature of smoking versus never smoking for stage 1 HNSCC is illustrated in Table 4.

### 3.5. Evaluation of Gene Expression Signature Based on Human Papilloma Virus 16 Status with/without Correlation with Smoking in Head and Neck Squamous Cell Carcinomas Patients

**Figure 5:**
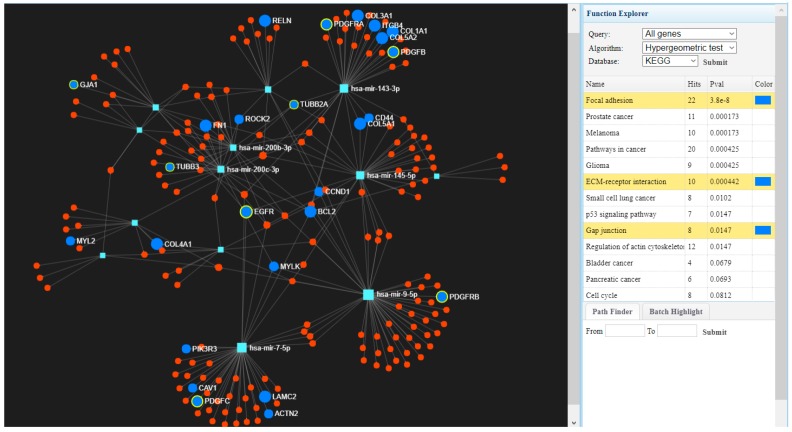
The interconnected genes with specific miRNAs using miRnet [22] involved in focal adhesion, ECM–receptor interaction or gap junction.

Using the same TCGA data, we performed a new analysis which allowed the identification of a signature composed of 2087 genes (1143 downregulated and 844 upregulated genes) that discriminates HPV16-induced HNSCC from their HPV-negative counterparts, comprising a patient cohort of 37 patients HPV-positive (HPV+) for subtype 16 and 72 HPV-negative (HPV−) patients. Using the miRnet data base, an analysis of the altered transcripts revealed the most relevant interconnected miRNAs and the most significantly altered pathways (Figure 5).

**Figure 6:**
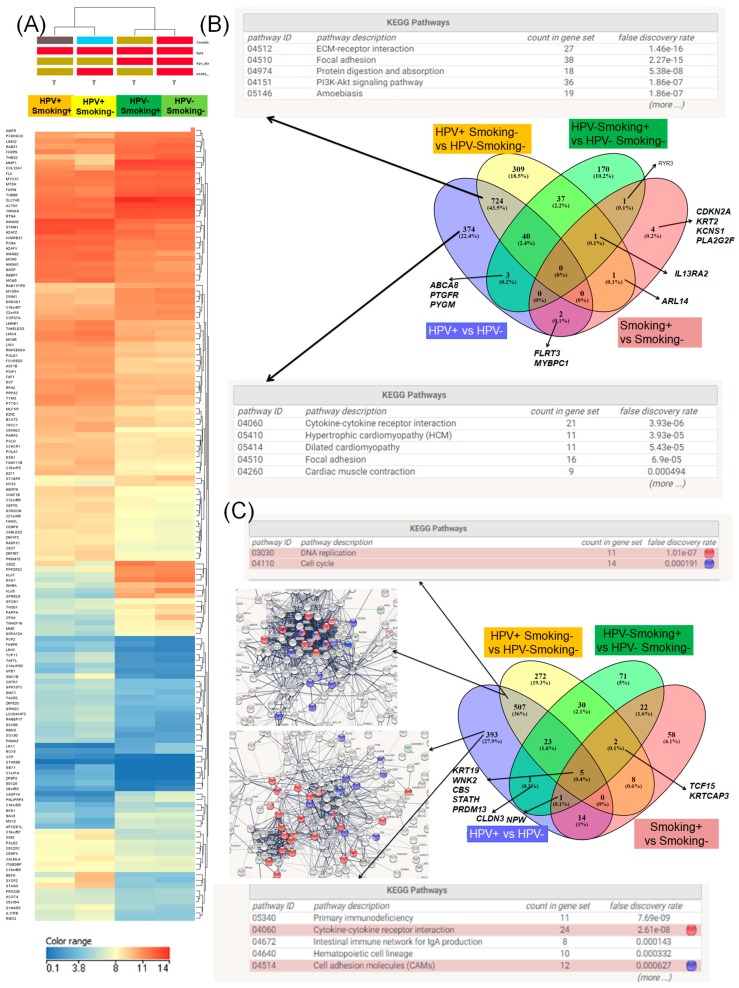
Gene expression signature based on HPV16 status with/without correlation with smoking in HNSCC patients. (**A**) Heat maps representing the expression level in the HNSCC patient group based on smoking and HPV status. For nonsmoking and HPV16-negative patients (Smoking− HPV−, *n* = 32), we had Smoking+ HPV− (*n* = 11), Smoking+ HPV+ (*n* = 11), Smoking− HPV+ (*n* = 11), in dark blue being presented the downregulated genes and in red those overexpressed genes, generated using Gene Spring version 13.0. (**B**) Venn diagram showing the differential signature in the case of the overexpressed genes highlighting the main altered pathways as displayed by KEGG classification. (**C**) Venn diagram to emphasize that the differential signature in the case of the overexpressed genes underlines the main altered pathways as obtained from String Network and KEGG (Kyoto Encyclopedia of Genes and Genomes) classification, with red dots showing the genes involved in cytokine–cytokine receptor interaction and blue dots the cell adhesion molecules.

A differential expression level comparison was performed, taking into consideration as reference group the nonsmoker patients negative for HPV (HPV− Smoke−), represented by 32 cases, while the other three analyzed groups were represented by smoking patients that were HPV-positive (HPV+ Smoke+, 11 cases), nonsmoking patients that were HPV-positive (HPV+ Smoke−), and smoking patients that were HPV-negative (Smoke+ HPV−, 11 patients). The heat map depicted in Figure 6A illustrates a specific signature in each analyzed group and in Table 5 being presented GO classification for the altered expression signature identified based on HPV-16 status.

Regarding the overexpressed genes, we observed a common signature represented by 724 genes in the case of the HPV+ versus HPV−, and the group (HPV+ Smoke−) versus (Smoke− HPV−); based on the KEGG classification, these genes are related to the ECM–receptor interaction, focal adhesion, and PI3K–Akt signaling (Figure 6B). We also identified 374 genes specifics for HPV+ versus HPV−, and 309 genes specific for the HPV+ Smoke− group (Figure 6B). In addition, 507 common genes were identified for the overexpressed genes involved in DNA replication and cell cycle, as obtained by KEGG classification (Figure 6C).

### 3.6. Survival Prognosis Analysis Related to Smoking Status in Head and Neck Squamous Cell Carcinomas Patients

The overall survival of HNSC patients related to three different groups based on smoking status: current smoker (*n* = 174), ex-smoker (*n* = 209), and never-smoking groups (*n* = 118) are presented in Figure S2. Also, survival analysis was performed in the case of HPV16+ (*n* = 72) versus HPV16– group (*n* = 37), observing a slightly increased survival rate in HPV− patients compared to HPV+ cases.

## 4. Discussion

The HNSCC disease etiology is complex, being related to genetic background and exposome, where smoking and viral infection are two important players in its causality [3,18,23,24,25,26,27]. HPV and smoking converge in more aggressive diseases through complex altered pathways (particularly those related to xenobiotic metabolism [23,28,29]) as observed in the presented data, with potentially important clinical implications. At the same time, smoking patients have a reduced overall survival when compared to non-smoking groups [30,31]; in our case, we can observe a slightly increased survival rate in the non-smoking group, with no statistical significance. The study of Osazuwa–Peters et al. shows that the survival rate is almost double in the non-smoking versus smoking group with HNSCC [32].

The overall variation in gene expression profiles for patients who quit smoking versus those who never smoked, and current smokers versus those who quit, was different when comparing tumors with normal tumor adjacent samples. The most significant differences were observed in the case of smoking versus never smoking. These observations are sustained by similar studies [33,34,35]. A set of 49 differentially expressed genes were detected based on smoking status, targeting NFkB-related pathways [36]. In comprehensive genomic characterization, we showed that most of the altered genes are related to the regulation of mutated TP53 and cell cycle progression [37]. A study similar to ours emphasized the important role of xenobiotic metabolizing enzymes in several cancer types like bladder [38] and lung cancer [39] or leukemia [40]; cytochrome (CY) P450 enzymes such as CYP1A1 are activated in the case of the smoking group as compared to never smokers [41]. Xenobiotic metabolizing enzymes CYP1A1 and CYP1B1 were observed also in a cellular model of oral leukoplakia [42]. The metabolic detoxification pathways have an important role in chemotherapeutics metabolism [42], affecting the response to therapy in smoking groups. The negative effects can be counteracted by chemopreventive agents [43,44,45,46].

Our study demonstrated the complex biologic effects of smoking through the analysis related to the effect of smoking on HNSCC stages, particularly for stage 1, emphasizing the altered pathways leading to carcinogenesis. In the case of gene expression signatures in smoking versus never smoking for stage 1 HNSCC patients, using the PantherDB online tool we observed an important number of representative transcripts that are responsible for biological adhesion, including for early stages (*DLL3*, *CDH17*, *TINAGL1*, *STRC*, *PCDHAC2*, *PCDHB13*, *TNR*, *PCDHGB6*, *PCDHGA9*, *PLXNB3*). The same analysis identified 32 downregulated genes related to cell cycle regulation. A cell culture-based study on human placental cells using cigarette smoke extracts showed alterations in cell cycle, cell migration, and endocrine activity [44,47], sustaining our findings. Alterations of these vital genes denote a frequent mechanism essential for the susceptibility to a variety of smoking-induced diseases [48]. These adhesion molecules are retrieved in the circulatory system and not only at tumor sites, especially in advanced stages [49,50,51]. These adhesion effectors and angiogenic markers could thus be used as biomarkers of invasion and metastasis [49,50,52,53,54,55]. Immune and inflammation-related genes may provide a better understanding of the mechanisms through which tobacco smoking causes disease [56], as well as the possible benefits of immune agonist therapy. A previous study showed that tumors with a genetic smoking pattern had decreased immune infiltration, connected with an unfavorable survival rate [57]. It was shown that the circulating immune markers of inflammation could mirror the overall immune and inflammatory cancer-promoting microenvironment [58,59], and may suggest probable etiologic mechanisms related to smoking-induced diseases, particularly in HNSCC. In our case, the altered immune and inflammatory response genes for stage I smoking vs. nonsmoking identified a panel of 13 genes obtained by PantherDB classification on biological processes (*CCL26*, *TRAF5*, *XCL1*, *BLK*, *DLL3*, *MAPK8IP2*, *CD1B*, *CD1E*, *CRYAB*, *GPX3*, *CCL22*, *LHX4*, *ULBP1*). Smoking also affects immunity in the oral cavity and promotes oral cavity diseases, including oral cancer [59]; hence, there is no doubt that immune microenvironment of HNSCC significantly affects the response to therapy [60].

The global gene expression signatures show the interaction between genetics and exposure characteristics, making the subtraction of a single agent effect related to HNSCC very difficult; in spite of this we were able to demonstrate that the transcriptomic pattern is highly influenced by tobacco smoking and HPV status. In the case of HPV+ HNSCCs, the mutational and transcriptomic pattern appeared similar to that of cervical cancer, with a higher mutation incidence in the PI3K pathway and DNA repair genes [61]. For transcriptomic pattern, altered genes implicated in cell cycle, apoptosis, inflammatory response, DNA replication and repair, or other important transcription factors involved in transcription regulation were revealed [47]. Gene expression characterization in HPV+ tumors can be used to predict response to therapy, and this information could be used for better tailored therapies [62]. Our data sustain the idea that HPV-related HNSCC represents a distinct entity, and that current treatment options are not responding to the needs of these patients [47,61], proving the necessity for routine testing of HPV in clinical practice [63] and at the same time underlining the importance of patient stratification based on smoking status or HPV infection.

## 5. Conclusions

In conclusion, we demonstrated that smoking and HPV infection make important contributions to the HNSCC genomic portrait; this information can be translated into the creation of better-tailored therapies. A part of the gene expression alteration pattern was the reversible signature related mainly to the metabolism of xenobiotics by cytochrome P450, but some genes remained altered even after quitting smoking. This study emphasizes the utility of HNSCC classification based on smoking status in the management of cancer risk and also in establishing therapeutic options based on the many altered cell signaling pathways that we identified (metabolic detoxification pathways, adhesion cell signaling, or immune and inflammation pathways).

Our pathway analysis was able to identify the most relevant gene expression signature for the smoking HNSCC patients related to xenobiotic metabolizing enzymes, which might affect the response to therapy. These data support the fact that smoking is a major risk factor for HNSCC outcomes and that smoking cessation therapy should be a part of standard HNSCC care. From a research viewpoint, these results emphasize the importance of environmental toxic agent exposure in corroboration with genetic background.

Since a wide range of factors affect gene expression, it is very likely that not all relevant gene transcripts were identified, and some genes with altered expression levels may not have been confirmed; however, these data represent an important starting point for new investigations. Future studies could examine other complexities of the transcriptome in relation with other environmental carcinogens. Regarding the exposome, is difficult to analyze single exposures due to the fact that for most cases a co-occurrence of toxic elements is observed.

## Figures and Tables

**Table 1:** Succinct presentation of the TCGA characteristics for patients diagnosed with HNSCCs used for gene expression analysis. Head and neck squamous cell carcinomas: HNSCCs, T: tumor, N: node, M0 no metastasis, Mx: metastases presence; HPV: human papilloma virus; TCGA: The Cancer Genome Atlas.

Clinical Parameters		Patients (*n* = 519)	Females/Males
Sex	Females	134	
	Males	374	
	Undeclared	11	
Age	Median, range	61, 19–90	
	Median, range males	59, 19–88	
	Median, range females	64.5, 24–90	
	Undeclared	11	
Clinical stage	1	20	9/11
	2	98	33/65
	3	101	26/75
	4	275	64/211
	Unknown	25	
Clinical TNM	T1N0M0	18	7/11
	T1N1M0	5	2/3
	T1N2M0	7	1/6
	T1N2M1	1	-/1
	T1NxM0	2	2/-
	T1N0M0	96	33/63
	T1N0Mx	1	-/1
	T2N1M0	12	3/9
	T2N1Mx	1	1/-
	T2N2M0	33	6/27
	T2N2M1	1	-/1
	T2N2Mx	3	-/3
	T1N3M0	1	-/1
	T2NxM0	1	-/1
	T3N0M0	58	15/43
	T3N0Mx	1	1/-
	T2N1M0	20	4/16
	T3N1M1	1	1/-
	T3N2M0	46	8/38
	T3N2M1	1	1/-
	T3N2Mx	1	-/1
	T3N3M0	1	-/1
	T3NxM0	2	-/2
	T4N0M0	66	17/49
	T4N0Mx	1	1/-
	T4N1M0	39	13/26
	T4N2M0	62	14/48
	T4N2Mx	1	-/1
	T4N3M0	7	2/5
	T4NxM0	3	-/3
	TxN1M0	1	-/1
	TxN2M0	1	-/1
	TxNxMx	9	-/9
	Unknown	16	
Smoking history	Smoker	174	35/139
	Reformed smoker <15 years	134	26/108
	Reformed smoker >15 years	72	19/53
	Reformed, unknown years	2	0/2
	Lifelong non-smoker	114	49/65
	Unknown	23	
Anatomic neoplasm subdivision	Alveolar ridge	18	
	Base of tongue	27	
	Buccal mucosa	20	
	Floor of mouth	60	
	Hard palate	7	
	Hypopharynx	9	
	Larynx	114	
	Lip	3	
	Oral cavity	71	
	Oral tongue	127	
	Oropharynx	10	
	Tonsil	42	
	Unknown	11	
HPV	Positive	72	
	Negative	37	
	Unknown	410	

**Table 2:** Genes with altered expression levels, based on a fold change (FC) ± 2, *p*-value ≤ 0.05 for smoking versus never smoking HNSCC patients.

Gene	FC (abs)	*p*-Value	Regulation	Gene	FC (abs)	*p*-Value	Regulation
*CDKN2A*	−2.59737	0.000603	Down	*EPHA7*	2.39461	0.000111	Up
*RYR3*	−2.35924	1.45 × 10^−6^	Down	*psiTPTE22*	2.394392	6.22 × 10^−6^	Up
*KRT2*	−2.31761	0.002097	Down	*POU6F2*	2.393242	3.2 × 10^−6^	Up
*KCNS1*	−2.31495	0.000321	Down	*SOHLH1*	2.390684	0.000256	Up
*MYBPC1*	−2.10091	0.046549	Down	*LTF*	2.380863	0.012372	Up
*ARL14*	−2.08283	0.000138	Down	*MLXIPL*	2.373807	8 × 10^−6^	Up
*IL13RA2*	−2.05906	8.82 × 10^−5^	Down	*GLI2*	2.372254	6.52 × 10^−7^	Up
*FLRT3*	−2.02315	0.002341	Down	*NLGN4Y*	2.358872	0.00092	Up
*PLA2G2F*	−2.01307	0.002746	Down	*PAK7*	2.355639	6.22 × 10^−6^	Up
*NTS*	5.224654	8 × 10^−6^	Up	*FIBCD1*	2.345015	0.000181	Up
*RPS4Y1*	4.924049	7.27 × 10^−5^	Up	*GATA4*	2.343389	0.000117	Up
*UGT1A6*	4.505707	3.3 × 10^−7^	Up	*PANX2*	2.319508	1.31 × 10^−6^	Up
*UPK1B*	4.336869	4.15 × 10^−5^	Up	*PCYT1B*	2.314619	3.29 × 10^−5^	Up
*MGST1*	4.296636	4.68 × 10^−7^	Up	*FGF19*	2.31125	3.18 × 10^−5^	Up
*CYP1A1*	4.269958	5.3 × 10^−13^	Up	*SLC44A4*	2.29202	0.000356	Up
*C20orf114*	3.985658	0.001123	Up	*SCN2A*	2.28985	0.00011	Up
*SCGB3A1*	3.985451	3.93 × 10^−5^	Up	*PROM1*	2.2842	0.001556	Up
*GPR15*	3.913692	2.69 × 10^−15^	Up	*CYorf15A*	2.257771	0.002582	Up
*DDX3Y*	3.748438	0.000214	Up	*TFPI2*	2.25193	0.000222	Up
*MUC5B*	3.573662	0.001207	Up	*MSI1*	2.249305	2.06 × 10^−5^	Up
*CNNM1*	3.508977	3.02 × 10^−8^	Up	*ADD2*	2.237741	6.45 × 10^−5^	Up
*CES1*	3.41544	1.21 × 10^−5^	Up	*ALDH1A1*	2.234898	0.000377	Up
*PRAME*	3.289014	0.000277	Up	*ERN2*	2.210954	0.000992	Up
*GPX2*	3.252181	3.95 × 10^−6^	Up	*LGI3*	2.206128	0.000183	Up
*CYP26A1*	3.241253	2.07 × 10^−5^	Up	*PRKY*	2.206114	0.002395	Up
*NR5A1*	3.186291	1.24 × 10^−5^	Up	*SALL1*	2.200458	8.32 × 10^−6^	Up
*PPP1R1B*	3.18047	1.38 × 10^−5^	Up	*TBX5*	2.194891	8.95 × 10^−6^	Up
*FGFBP2*	3.156294	2.95 × 10^−6^	Up	*HOXA4*	2.181679	4.15 × 10^−6^	Up
*SLC13A5*	3.142512	6.04 × 10^−6^	Up	*TNNI3*	2.18105	4.06 × 10^−5^	Up
*DMBT1*	3.102893	0.000721	Up	*PLUNC*	2.179797	0.009085	Up
*KRTCAP3*	3.077278	7.92 × 10^−12^	Up	*NGB*	2.177274	0.000323	Up
*BPIL1*	3.011847	0.002582	Up	*FOLR1*	2.175127	0.000402	Up
*UGT1A8*	3.00819	1.43 × 10^−5^	Up	*GDA*	2.163271	0.00378	Up
*PTH2R*	2.849531	2.55 × 10^−5^	Up	*AKR1C3*	2.150982	8.66 × 10^−5^	Up
*KRT19*	2.812564	0.001704	Up	*AZGP1*	2.142351	0.004176	Up
*GAL*	2.792954	6.04 × 10^−6^	Up	*CCNA1*	2.138528	0.001642	Up
*EIF1AY*	2.761805	0.001147	Up	*PCDH19*	2.131033	0.000887	Up
*WNK2*	2.756387	3.74 × 10^−5^	Up	*GJB7*	2.128482	0.000382	Up
*B4GALNT4*	2.731352	5.73 × 10^−7^	Up	*WDR72*	2.119921	0.003053	Up
*RAB3B*	2.721629	2.09 × 10^−7^	Up	*CLDN8*	2.118758	0.001164	Up
*FAM132A*	2.67193	2.95 × 10^−6^	Up	*CBS*	2.117995	2.03 × 10^−5^	Up
*HOXA7*	2.666479	3.29 × 10^−5^	Up	*MSMB*	2.117211	0.003146	Up
*PIGR*	2.652387	0.004096	Up	*CFTR*	2.112852	0.00034	Up
*BCHE*	2.639082	3.2 × 10^−6^	Up	*NTRK2*	2.112379	0.001737	Up
*UGT8*	2.635306	5.71 × 10^−5^	Up	*FGF13*	2.108137	2.2 × 10^−5^	Up
*USP9Y*	2.629508	0.001642	Up	*RPL39L*	2.085114	5.8 × 10^−6^	Up
*PDIA2*	2.613751	2.12 × 10^−6^	Up	*SLC29A4*	2.079458	4.68 × 10^−7^	Up
*ZFY*	2.558155	0.0013	Up	*ADH7*	2.07414	0.012696	Up
*CALB1*	2.545223	0.002413	Up	*PIWIL2*	2.068965	0.000399	Up
*AKR1C1*	2.540783	7.2 × 10^−6^	Up	*CYP1B1*	2.057724	8.81 × 10^−5^	Up
*UTY*	2.533913	0.00193	Up	*CPNE7*	2.055357	1.57 × 10^−6^	Up
*ATP13A5*	2.530633	0.00011	Up	*BRDT*	2.041222	0.001179	Up
*SLC5A12*	2.504015	2.95 × 10^−6^	Up	*CHGA*	2.033392	2.12 × 10^−5^	Up
*FOXJ1*	2.491234	0.000516	Up	*ABO*	2.032663	0.001283	Up
*PRDM13*	2.479601	4.03 × 10^−7^	Up	*STATH*	2.023424	0.020511	Up
*HORMAD1*	2.437306	0.001219	Up	*SCN9A*	2.018196	0.00048	Up
*UCHL1*	2.434416	7.96 × 10^−6^	Up	*ADAMTS20*	2.011134	0.000245	Up
*NPW*	2.41423	6.04 × 10^−6^	Up	*RBM11*	2.010622	4.12 × 10^−5^	Up
*PNCK*	2.396283	8.83 × 10^−5^	Up	*ZNF556*	2.009657	1.24 × 10^−5^	Up
				*TCF15*	2.007499	4.31 × 10^−5^	Up

**Table 3:** Gene ontology (GO) classification based on the gene expression signature in smoking versus never-smoking HNSCC patients using the PantherDB online tool [21].

Ontology Function	Type	No. Molecules	Percent (%)
Molecular function	binding (GO:0005488)	33	36.7%
	catalytic activity (GO:0003824)	30	33.3%
	transporter activity (GO:0005215)	14	15.6%
	receptor activity (GO:0004872)	4	4.4%
	signal transducer activity (GO:0004871)	4	4.4%
	structural molecule activity (GO:0005198)	3	3.3%
	translation regulator activity (GO:0045182)	1	1.1%
	antioxidant activity (GO:0016209)	1	1.1%
Biological process	cellular process (GO:0009987)	48	28.1%
	metabolic process (GO:0008152)	32	18.7%
	biological regulation (GO:0065007)	23	13.5%
	developmental process (GO:0032502)	16	9.4%
	response to stimulus (GO:0050896)	15	8.8%
	multicellular organismal process (GO:0032501)	13	7.6%
	localization (GO:0051179)	8	4.7%
	cellular component organization or biogenesis (GO:0071840)	7	4.1%
	biological adhesion (GO:0022610)	4	2.3%
	locomotion (GO:0040011)	3	1.8%
	immune system process (GO:0002376)	1	0.6%
	reproduction (GO:0000003)	1	0.6%
Protein class	transporter (PC00227)	10	13.5%
	hydrolase (PC00121)	9	12.2%
	oxidoreductase (PC00176)	8	10.8%
	transcription factor (PC00218)	8	10.8%
	nucleic acid binding (PC00171)	7	9.5%
	signaling molecule (PC00207)	6	8.1%
	transferase (PC00220)	5	6.8%
	enzyme modulator (PC00095)	5	6.8%
	receptor (PC00197)	3	4.1%
	extracellular matrix protein (PC00102)	2	2.7%
	cytoskeletal protein (PC00085)	2	2.7%
	transfer/carrier protein (PC00219)	2	2.7%
	cell junction protein (PC00070)	2	2.7%
	lyase (PC00144)	1	1.4%
	calcium-binding protein (PC00060)	1	1.4%
	defense/immunity protein (PC00090)	1	1.4%
	membrane traffic protein (PC00150)	1	1.4%
	isomerase (PC00135)	1	1.4%

**Table 4:** Gene ontology (GO) classification based on the gene expression signature in smoking versus never smoking for stage 1 HNSCC patients using the PantherDB online tool, showing the maximum effect of smoking and the minimum effect of tumor environment.

Ontology Function	Type	No. Molecules	Percent (%)
Molecular function	binding (GO:0005488)	1433	37.5%
	catalytic activity (GO:0003824)	1262	33.0%
	transporter activity (GO:0005215)	429	11.2%
	receptor activity (GO:0004872)	255	6.7%
	signal transducer activity (GO:0004871)	231	6.0%
	structural molecule activity (GO:0005198)	157	4.1%
	antioxidant activity (GO:0016209)	23	0.6%
	translation regulator activity (GO:0045182)	17	0.4%
	channel regulator activity (GO:0016247)	13	0.3%
Biological process	cellular process (GO:0009987)	2479	29.5%
	metabolic process (GO:0008152)	1589	18.9%
	biological regulation (GO:0065007)	911	10.9%
	response to stimulus (GO:0050896)	696	8.3%
	localization (GO:0051179)	583	6.9%
	cellular component organization or biogenesis (GO:0071840)	581	6.9%
	developmental process (GO:0032502)	534	6.4%
	multicellular organismal process (GO:0032501)	523	6.2%
	immune system process (GO:0002376)	146	1.7%
	locomotion (GO:0040011)	120	1.4%
	biological adhesion (GO:0022610)	110	1.3%
	reproduction (GO:0000003)	83	1.0%
	rhythmic process (GO:0048511)	32	0.4%
	cell killing (GO:0001906)	6	0.1%
Protein class	nucleic acid binding (PC00171)	565	15.2%
	transcription factor (PC00218)	504	13.5%
	hydrolase (PC00121)	410	11.0%
	receptor (PC00197)	355	9.5%
	transporter (PC00227)	269	7.2%
	signaling molecule (PC00207)	251	6.7%
	transferase (PC00220)	240	6.4%
	cytoskeletal protein (PC00085)	192	5.2%
	enzyme modulator (PC00095)	179	4.8%
	oxidoreductase (PC00176)	149	4.0%
	extracellular matrix protein (PC00102)	84	2.3%
	membrane traffic protein (PC00150)	83	2.2%
	ligase (PC00142)	79	2.1%
	calcium-binding protein (PC00060)	72	1.9%
	structural protein (PC00211)	63	1.7%
	isomerase (PC00135)	42	1.1%
	lyase (PC00144)	36	1.0%
	defense/immunity protein (PC00090)	35	0.9%
	cell adhesion molecule (PC00069)	31	0.8%
	cell junction protein (PC00070)	31	0.8%
	chaperone (PC00072)	24	0.6%
	transfer/carrier protein (PC00219)	19	0.5%
	transmembrane receptor regulatory/adaptor protein (PC00226)	11	0.3%

**Table 5:** Gene ontology (GO) classification based on the gene expression signature in HPV16-positive versus HPV16-negative patients using the PantherDB online tool [21].

Ontology Function	Type	No. Molecules	Percent (%)
Molecular function	binding (GO:0005488)	6900	41.1%
	catalytic activity (GO:0003824)	5156	30.7%
	transporter activity (GO:0005215)	1474	8.8%
	receptor activity (GO:0004872)	1450	8.6%
	signal transducer activity (GO:0004871)	895	5.3%
	structural molecule activity (GO:0005198)	826	4.9%
	translation regulator activity (GO:0045182)	53	0.3%
	antioxidant activity (GO:0016209)	23	0.1%
	binding (GO:0005488)	6900	41.1%
Biological process	cellular process (GO:0009987)	10968	28.1%
	metabolic process (GO:0008152)	6589	16.9%
	biological regulation (GO:0065007)	4065	10.4%
	response to stimulus (GO:0050896)	3467	8.9%
	developmental process (GO:0032502)	3319	8.5%
	multicellular organismal process (GO:0032501)	2954	7.6%
	cellular component organization or biogenesis (GO:0071840)	2626	6.7%
	localization (GO:0051179)	2125	5.4%
	immune system process (GO:0002376)	975	2.5%
	biological adhesion (GO:0022610)	898	2.3%
	locomotion (GO:0040011)	668	1.7%
	reproduction (GO:0000003)	289	0.7%
	rhythmic process (GO:0048511)	28	0.1%
	growth (GO:0040007)	27	0.1%
Protein class	hydrolase (PC00121)	2235	13.2%
	nucleic acid binding (PC00171)	1676	9.9%
	signaling molecule (PC00207)	1598	9.5%
	transcription factor (PC00218)	1551	9.2%
	enzyme modulator (PC00095)	1462	8.7%
	receptor (PC00197)	1324	7.8%
	cytoskeletal protein (PC00085)	987	5.9%
	transferase (PC00220)	987	5.9%
	transporter (PC00227)	977	5.8%
	oxidoreductase (PC00176)	667	4.0%
	extracellular matrix protein (PC00102)	632	3.7%
	cell adhesion molecule (PC00069)	560	3.3%
	calcium-binding protein (PC00060)	362	2.1%
	membrane traffic protein (PC00150)	331	2.0%
	cell junction protein (PC00070)	307	1.8%
	defense/immunity protein (PC00090)	278	1.6%
	ligase (PC00142)	185	1.1%
	structural protein (PC00211)	173	1.0%
	chaperone (PC00072)	157	0.9%
	transmembrane receptor regulatory/adaptor protein (PC00226)	147	0.9%
	lyase (PC00144)	134	0.8%
	isomerase (PC00135)	66	0.4%
	transfer/carrier protein (PC00219)	63	0.4%

## Supplementary Materials

The following are available online at http://www.mdpi.com/1660-4601/15/7/1558/s1.

## References

[1] Abrahao R., Anantharaman D., Gaborieau V., Abedi-Ardekani B., Lagiou P., Lagiou A., Ahrens W., Holcatova I., Betka J., Merletti F. (2018). The influence of smoking, age and stage at diagnosis on the survival after larynx, hypopharynx and oral cavity cancers in Europe: The ARCAGE study. Int. J. Cancer.

[2] Irimie A.I., Braicu C., Cojocneanu-Petric R., Berindan-Neagoe I., Campian R.S. (2015). Novel technologies for oral squamous carcinoma biomarkers in diagnostics and prognostics. Acta Odontol. Scand..

[3] Giraldi L., Leoncini E., Pastorino R., Wunsch-Filho V., de Carvalho M., Lopez R., Cadoni G., Arzani D., Petrelli L., Matsuo K. (2017). Alcohol and cigarette consumption predict mortality in patients with head and neck cancer: A pooled analysis within the International Head and Neck Cancer Epidemiology (INHANCE) Consortium. Ann. Oncol..

[4] Irimie A.I., Zimta A.A., Ciocan C., Mehterov N., Dudea D., Braicu C., Berindan-Neagoe I. (2018). The Unforeseen Non-Coding RNAs in Head and Neck Cancer. Genes.

[5] Ferlay J., Shin H.R., Bray F., Forman D., Mathers C., Parkin D.M. (2010). Estimates of worldwide burden of cancer in 2008: GLOBOCAN 2008. Int. J. Cancer.

[6] Salyakina D., Tsinoremas N.F. (2016). Non-coding RNAs profiling in head and neck cancers. NPJ Genom. Med..

[7] Irimie A.I., Braicu C., Sonea L., Zimta A.A., Cojocneanu-Petric R., Tonchev K., Mehterov N., Diudea D., Buduru S., Berindan-Neagoe I. (2017). A looking-glass of non-coding RNAs in oral cancer. Int. J. Mol. Sci..

[8] Irimie A.I., Sonea L., Jurj A., Mehterov N., Zimta A.A., Budisan L., Braicu C., Berindan-Neagoe I. (2017). Future trends and emerging issues for nanodelivery systems in oral and oropharyngeal cancer. Int. J. Nanomed..

[9] Feng H.-M., Zhao Y., Zhang J.-P., Zhang J.-H., Jiang P., Li B., Wang C. (2018). Expression and potential mechanism of metabolism-related genes and CRLS1 in non-small cell lung cancer. Oncol. Lett..

[10] Wang T.H., Hsia S.M., Shih Y.H., Shieh T.M. (2017). Association of smoking, alcohol use, and betel quid chewing with epigenetic aberrations in cancers. Int. J. Mol. Sci..

[11] Leemans C.R., Snijders P.J.F., Brakenhoff R.H. (2018). The molecular landscape of head and neck cancer. Nat. Rev. Cancer.

[12] Vineis P., Chadeau-Hyam M., Gmuender H., Gulliver J., Herceg Z., Kleinjans J., Kogevinas M., Kyrtopoulos S., Nieuwenhuijsen M., Phillips D.H. (2017). The exposome in practice: Design of the EXPOsOMICS project. Int. J. Hyg. Environ. Health.

[13] Van Breda S.G.J., Wilms L.C., Gaj S., Jennen D.G.J., Briedé J.J., Kleinjans J.C.S., de Kok T.M.C.M. (2015). The exposome concept in a human nutrigenomics study: Evaluating the impact of exposure to a complex mixture of phytochemicals using transcriptomics signatures. Mutagenesis.

[14] Tang Z., Li C., Kang B., Gao G., Li C., Zhang Z. (2017). GEPIA: A web server for cancer and normal gene expression profiling and interactive analyses. Nucleic Acids Res..

[15] Singh R.K., Sivabalakrishnan M. (2015). Feature selection of gene expression data for cancer classification: A review. Procedia Comput. Sci..

[16] Zhang X., Cha I.-H., Kim K.-Y. (2017). Highly preserved consensus gene modules in human papilloma virus 16 positive cervical cancer and head and neck cancers. Oncotarget.

[17] Braicu C., Catana C., Calin G.A., Berindan-Neagoe I. (2014). NCRNA combined therapy as future treatment option for cancer. Curr. Pharm. Des..

[18] UCSC Genome Browser. https://genome.ucsc.edu.

[19] STRING: Functional Protein Association Network, Version 10.5. https://string-db.org.

[20] KEGG Pathway Maps. http://www.genome.jp/kegg/pathway.html.

[21] Panther Clasiffication System. http://www.pantherdb.org.

[22] miRNet-Network-Based Visual Analysis of miRNAs, Targets and Functions. http://www.mirnet.ca/faces/home.xhtml.

[23] Campbell J.D., Yau C., Bowlby R., Liu Y., Brennan K., Fan H., Taylor A.M., Wang C., Walter V., Akbani R. (2018). Genomic, pathway network, and immunologic features distinguishing squamous carcinomas. Cell Rep..

[24] Ferketich A.K., Niland J.C., Mamet R., Zornosa C., D’Amico T.A., Ettinger D.S., Kalemkerian G.P., Pisters K.M., Reid M.E., Otterson G.A. (2013). Smoking status and survival in the national comprehensive cancer network non-small cell lung cancer cohort. Cancer.

[25] Bryant A., Cerfolio R.J. (2007). Differences in epidemiology, histology, and survival between cigarette smokers and never-smokers who develop non-small cell lung cancer. Chest.

[26] Koshiaris C., Aveyard P., Oke J., Ryan R., Szatkowski L., Stevens R., Farley A. (2017). Smoking cessation and survival in lung, upper aero-digestive tract and bladder cancer: Cohort study. Br. J. Cancer.

[27] Pierce J.P., Patterson R.E., Senger C.M., Flatt S.W., Caan B.J., Natarajan L., Nechuta S.J., Poole E.M., Shu X.-O., Chen W.Y. (2014). Lifetime cigarette smoking and breast cancer prognosis in the after breast cancer pooling project. JNCI J. Nat. Cancer Inst..

[28] Champion M., Brennan K., Croonenborghs T., Gentles A.J., Pochet N., Gevaert O. (2018). Module analysis captures pancancer genetically and epigenetically deregulated cancer driver genes for smoking and antiviral response. EBioMedicine.

[29] Yavorski J.M., Blanck G. (2016). Smoking correlates with increased cytoskeletal protein-related coding region mutations in the lung and head and neck datasets of the cancer genome atlas. Physiol. Rep..

[30] Hoffmann M., Quabius E.S., Tribius S., Hebebrand L., Gorogh T., Halec G., Kahn T., Hedderich J., Rocken C., Haag J. (2013). Human papillomavirus infection in head and neck cancer: The role of the secretory leukocyte protease inhibitor. Oncol. Rep..

[31] Quabius E.S., Moller P., Haag J., Pfannenschmidt S., Hedderich J., Gorogh T., Rocken C., Hoffmann M. (2014). The role of the antileukoprotease SLPI in smoking-induced human papillomavirus-independent head and neck squamous cell carcinomas. Int. J. Cancer.

[32] Osazuwa-Peters N., Adjei Boakye E., Chen B.Y., Tobo B.B., Varvares M.A. (2018). Association Between Head and Neck Squamous Cell Carcinoma Survival, Smoking at Diagnosis, and Marital Status. JAMA Otolaryngol..

[33] Rudin C.M., Avila-Tang E., Harris C.C., Herman J.G., Hirsch F.R., Pao W., Schwartz A.G., Vahakangas K.H., Samet J.M. (2009). Lung cancer in never smokers: Molecular profiles and therapeutic implications. Clin. Cancer Res..

[34] Delagranda A., Leterme G., Chirpaz E., Ferdynus C., Fernandez C., Rubin F. (2018). Epidemiological features of cancers of the oral cavity, oropharynx, hypopharynx and larynx cancer in Reunion Island. Eur. Ann. Otorhinolaryngol. Head Neck Dis..

[35] Cufari M.E., Proli C., De Sousa P., Raubenheimer H., Al Sahaf M., Chavan H., Shedden L., Niwaz Z., Leung M., Nicholson A.G. (2017). Increasing frequency of non-smoking lung cancer: Presentation of patients with early disease to a tertiary institution in the UK. Eur. J. Cancer.

[36] Farshadpour F., Roepman P., Hordijk G.J., Koole R., Slootweg P.J. (2012). A gene expression profile for non-smoking and non-drinking patients with head and neck cancer. Oral Dis..

[37] Cancer Genome Atlas Network (2015). Comprehensive genomic characterization of head and neck squamous cell carcinomas. Nature.

[38] Rouissi K., Ouerhani S., Hamrita B., Bougatef K., Marrakchi R., Cherif M., Ben Slama M.R., Bouzouita M., Chebil M., Ben Ammar Elgaaied A. (2011). Smoking and polymorphisms in xenobiotic metabolism and DNA repair genes are additive risk factors affecting bladder cancer in Northern Tunisia. Pathol. Oncol. Res..

[39] Ihsan R., Chauhan P.S., Mishra A.K., Yadav D.S., Kaushal M., Sharma J.D., Zomawia E., Verma Y., Kapur S., Saxena S. (2011). Multiple analytical approaches reveal distinct gene-environment interactions in smokers and non smokers in lung cancer. PLoS ONE.

[40] Chauhan P.S., Ihsan R., Mishra A.K., Yadav D.S., Saluja S., Mittal V., Saxena S., Kapur S. (2012). High order interactions of xenobiotic metabolizing genes and P53 codon 72 polymorphisms in acute leukemia. Environ. Mol. Mutagen..

[41] Petros W.P., Younis I.R., Ford J.N., Weed S.A. (2012). Effects of tobacco smoking & nicotine on cancer treatment. Pharmacotherapy.

[42] Gümüş Z.H., Du B., Kacker A., Boyle J.O., Bocker J.M., Mukherjee P., Subbaramaiah K., Dannenberg A.J., Weinstein H. (2008). Effects of tobacco smoke on gene expression and cellular pathways in a cellular model of oral leukoplakia. Cancer Prev. Res..

[43] Irimie A.I., Braicu C., Zanoaga O., Pileczki V., Gherman C., Berindan-Neagoe I., Campian R.S. (2015). Epigallocatechin-3-gallate suppresses cell proliferation and promotes apoptosis and autophagy in oral cancer SSC-4 cells. Onco Targets Ther..

[44] Braicu C., Mehterov N., Vladimirov B., Sarafian V., Nabavi S.M., Atanasov A.G., Berindan-Neagoe I. (2017). Nutrigenomics in cancer: Revisiting the effects of natural compounds. Semin. Cancer Biol..

[45] Budisan L., Gulei D., Zanoaga O.M., Irimie A.I., Sergiu C., Braicu C., Gherman C.D., Berindan-Neagoe I. (2017). Dietary intervention by phytochemicals and their role in modulating coding and non-coding genes in cancer. Int. J. Mol. Sci..

[46] Kim C.W., Lee H.M., Lee K., Kim B., Lee M.Y., Choi K.C. (2017). Effects of cigarette smoke extracts on cell cycle, cell migration and endocrine activity in human placental cells. Reprod. Toxicol..

[47] Mirghani H., Ugolin N., Ory C., Lefevre M., Baulande S., Hofman P., St Guily J.L., Chevillard S., Lacave R. (2014). A predictive transcriptomic signature of oropharyngeal cancer according to HPV16 status exclusively. Oral Oncol..

[48] Scott D.A., Palmer R.M. (2003). The influence of tobacco smoking on adhesion molecule profiles. Tob. Induc. Dis..

[49] Zahra A., Rubab I., Malik S., Khan A., Khan M.J., Fatmi M.Q. (2018). Meta-Analysis of miRNAs and their involvement as biomarkers in oral cancers. BioMed Res. Int..

[50] Palmer R.M., Stapleton J.A., Sutherland G., Coward P.Y., Wilson R.F., Scott D.A. (2002). Effect of nicotine replacement and quitting smoking on circulating adhesion molecule profiles (sICAM-1, sCD44v5, sCD44v6). Eur. J. Clin. Investig..

[51] Scott D.A., Stapleton J.A., Palmer R.M., Wilson R.F., Sutherland G., Coward P.Y., Gustavsson G., Odell E.W., Poston R.N. (2000). Plasma concentrations of reputed tumor-associated soluble CD44 isoforms (v5 and v6) in smokers are dose related and decline on smoking cessation. Cancer Epidemiol. Biomark. Prev..

[52] Ionescu C., Braicu C., Chiorean R., Cojocneanu Petric R., Neagoe E., Pop L., Chira S., Berindan-Neagoe I. (2014). TIMP-1 expression in human colorectal cancer is associated with SMAD3 gene expression levels: A pilot study. JGLD.

[53] Braicu C., Tudoran O., Balacescu L., Catana C., Neagoe E., Berindan-Neagoe I., Ionescu C. (2013). The significance of PDGF expression in serum of colorectal carcinoma patients—Correlation with Duke’s classification. Can PDGF become a potential biomarker?. Chirurgia.

[54] Ionescu S., Barbu E., Ionescu C., Costache A., Balasoiu M. (2015). Giant gastrointestinal stromal tumor of the stomach. Rom. J. Morphol. Embryol..

[55] Scott D.A., Todd D.H., Coward P.Y., Wilson R.F., Odell E.W., Poston R.N., Matthews J.P., Palmer R.M. (2000). The acute influence of tobacco smoking on adhesion molecule expression on monocytes and neutrophils and on circulating adhesion molecule levels in vivo. Addict. Biol..

[56] Shiels M.S., Katki H.A., Freedman N.D., Purdue M.P., Wentzensen N., Trabert B., Kitahara C.M., Furr M., Li Y., Kemp T.J. (2014). Cigarette smoking and variations in systemic immune and inflammation markers. J. Natl. Cancer Inst..

[57] Mandal R., Şenbabaoğlu Y., Desrichard A., Havel J.J., Dalin M.G., Riaz N., Lee K.-W., Ganly I., Hakimi A.A., Chan T.A. (2016). The head and neck cancer immune landscape and its immunotherapeutic implications. JCI Insight.

[58] Desrichard A., Kuo F., Chowell D., Lee K.W., Riaz N., Wong R.J., Chan T.A., Morris L.G.T. (2018). Tobacco Smoking-Associated Alterations in the Immune Microenvironment of Squamous Cell Carcinomas. J. Natl. Cancer Inst..

[59] Lee J., Taneja V., Vassallo R. (2012). Cigarette smoking and inflammation: Cellular and molecular mechanisms. J. Dent. Res..

[60] Solomon B., Young R.J., Rischin D. (2018). Head and neck squamous cell carcinoma: Genomics and emerging biomarkers for immunomodulatory cancer treatments. Semin. Cancer Biol..

[61] Dok R., Nuyts S. (2016). HPV positive head and neck cancers: Molecular pathogenesis and evolving treatment strategies. Cancers.

[62] Thibodeau B.J., Geddes T.J., Fortier L.E., Ahmed S., Pruetz B.L., Wobb J., Chen P., Wilson G.D., Akervall J.A. (2015). Gene expression Characterization of HPV positive head and neck cancer to predict response to chemoradiation. Head Neck Pathol..

[63] Bishop J.A., Lewis J.S., Rocco J.W., Faquin W.C. (2015). HPV-related squamous cell carcinoma of the head and neck: An update on testing in routine pathology practice. Semin. Diagn. Pathol..

