# Supplementary material for: Differential Effect of Smoking on Gene Expression in Head and Neck Cancer Patients
Source: Int J Environ Res Public Health. 2018 Jul 23;15(7):1558. doi: 10.3390/ijerph15071558 (PMC6069101; doi:10.3390/ijerph15071558)
Supplement: Supplementary file 1 [file ijerph-15-01558-s001.pdf]

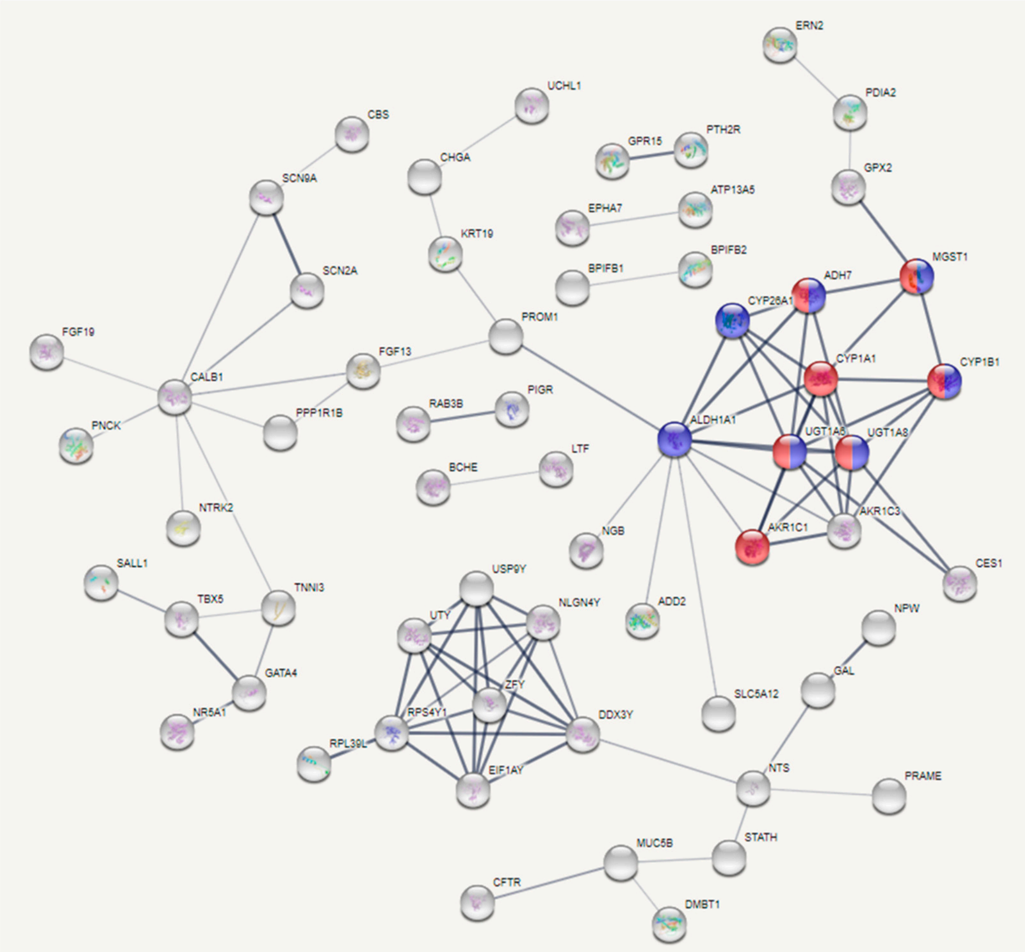

Functional enrichments in your network *Note: some enrichments may be expected here (why?)*

Biological Process (GO)			
pathway ID	*pathway description*	*count in gene set*	*false discovery rate*
GO:0001523	retinoid metabolic process	5	0.0189
GO:0006805	xenobiotic metabolic process	7	0.0189
GO:0007283	spermatogenesis	11	0.0189
GO:0007611	learning or memory	8	0.0189
GO:0007612	learning	7	0.0189
			(more ...)

KEGG Pathways			
pathway ID	*pathway description*	*count in gene set*	*false discovery rate*
00980	Metabolism of xenobiotics by cytochrome P450	7	1.59e-05
00140	Steroid hormone biosynthesis	6	4.54e-05
00830	Retinol metabolism	6	5.22e-05
05204	Chemical carcinogenesis	6	0.000131
00982	Drug metabolism - cytochrome P450	4	0.0184

Figure S1. String Network emphasizing the genes responsible for the metabolism of xenobiotics by cytochrome P450 (red dots) and the genes involved in xenobiotic metabolic processes (blue dots).

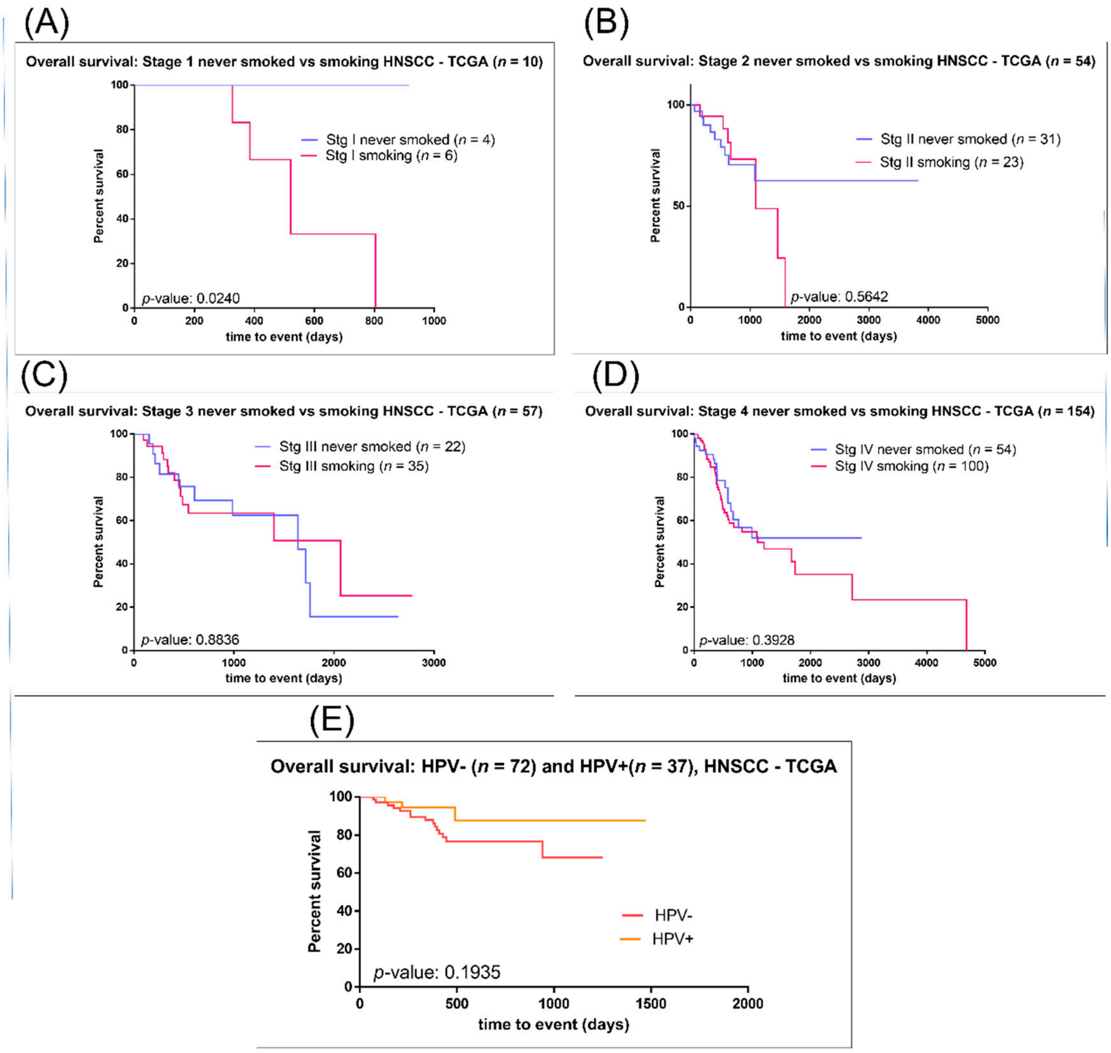

Figure S2. Overall survival of patients with HNSCCs based on smoking status (currently smoking, ex-smokers and never smoked), (**A**) for stage I; (**B**) stage II; (**C**) stage III; (**D**) stage IV and (**E**) related to HPV status (HPV−, HPV+).
